# Breast Implant-Associated Anaplastic Large Cell Lymphoma

**DOI:** 10.1155/2020/2157485

**Published:** 2020-05-28

**Authors:** P. L. Moori, F. Ibison, D. Jacob, J. Iddon

**Affiliations:** ^1^Department of Breast Surgery, Burnley General Hospital, Casterton Avenue, Burnley BB10 2PQ, UK; ^2^Department of Histopathology, Royal Blackburn Hospital, Haslingden Road, Blackburn BB2 3HH, UK

## Abstract

Breast implant-associated anaplastic large cell lymphoma (BIA-ALCL) is a rare, non-Hodgkin lymphoma which arises within the capsules of breast implants. These particular tumours have expression of CD30 and are negative for Anaplastic Lymphoma Kinase (ALK). Here, we report a case of BIA-ALCL in a 48-year-old woman post breast reconstruction. This case report is aimed at raising awareness and education on the significance of considering the development of BIA-ALCL in cases where cytology is negative and helping better understand this disease process.

## 1. Introduction

Breast implant-associated anaplastic large cell lymphoma (BIA-ALCL) is a recognised but rare entity, which was first reported in 1997 in association with saline breast implants [1]. Since first being described, approximately 400 cases have now been recounted within the literature [2]. Previous studies estimated the risk of BIA-ALCL as 1 in 300,000 breast implants. However, recent data from the Australia and New Zealand cohort of BIA-ALCL cases shows a higher risk estimate, with textured implants carrying significantly higher risk [3]. BIA-ALCL is a subtype of T-cell non-Hodgkin lymphoma, but its exact aetiology and pathogenesis are not yet understood [4]. Some concepts relate BIA-ALCL to implant biofilms, where bacteria attach to the implant surface and become surrounded by a layer of glycoprotein [5]. The glycoprotein layer makes the bacteria very difficult to treat, allowing the bacteria to lay quiescent for many years [6]. There are theories that a sustained T-cell immune response to the bacterial biofilm is what derives the progression of BIA-ALCL tumour cells; however, there is no fundamental evidence to support this. Patients with BIA-ALCL may present with various symptoms, the most common being breast swelling (>1 year postoperatively) and unilateral seroma formation between the capsule and implant surface [7]. Less frequently, the capsule may have localised thickening or a discrete mass. Although extremely rare, BIA-ALCL can occur bilaterally, and therefore, removal of both implants is advised. Cases of BIA-ALCL have been reported between 3 and 14 years postsurgery [8]. Early detection and management of BIA-ALCL, which is usually surgical, are essential as it can be curative, with a favourable prognosis [9]. We present a case of BIA-ALCL in a patient with negative aspiration cytology. This case is aimed at raising awareness and education on the significance of considering the development of BIA-ALCL in cases where cytology is negative and helping better understand this disease process.

## 2. Case Report

A 48-year-old woman underwent a bilateral mastectomy in 1997 followed by radiotherapy and chemotherapy for a BRCA-1-positive invasive ductal carcinoma. This was followed by a delayed bilateral breast reconstruction with implants in 2007 and later converted to latissimus dorsi flaps and implants (Nagor GFX 460 implants). Apart from an episode of pain in the left breast reconstruction in 2015, she did not present with any symptoms until June 2018, when she attended breast clinic with a few weeks' history of swelling in the right breast reconstruction. She denied any pain or trauma and was systemically well. On examination, there was no redness or tenderness and no palpable lymphadenopathy in the neck or axillae. The right breast reconstruction appeared slightly larger than the left and on palpation was tense, but not hard, when compared to the left side. An ultrasound scan demonstrated peri-implant fluid; the implant itself appeared intact. A fluid sample was aspirated and sent for microbiology and cytology in fixative. The fluid sample was reported locally as showing inflammatory cells, with no malignant cells identified. The microbiological microscopy, culture, and sensitivity testing of the seroma fluid were negative. The Haematological Malignancy Diagnostic Service (HMDS) in Leeds confirmed that there was no indication of lymphoma evident in this sample. It is notable that the fluid sample for cytology, adjusting for the usual fixative volume, seems to have been only 10 ml. No additional tests were performed on the cytology specimen due to the absence of a natural cell clot and insufficient fluid volume, creating the inability to produce an artificial clot or cell block for immunocytochemical studies. The cytology specimen was double-reported by HMDS Leeds and also considered negative by them. The “false negative” is considered most likely to be due to insufficient volume rather than diagnostic error.

**Figure 1:**
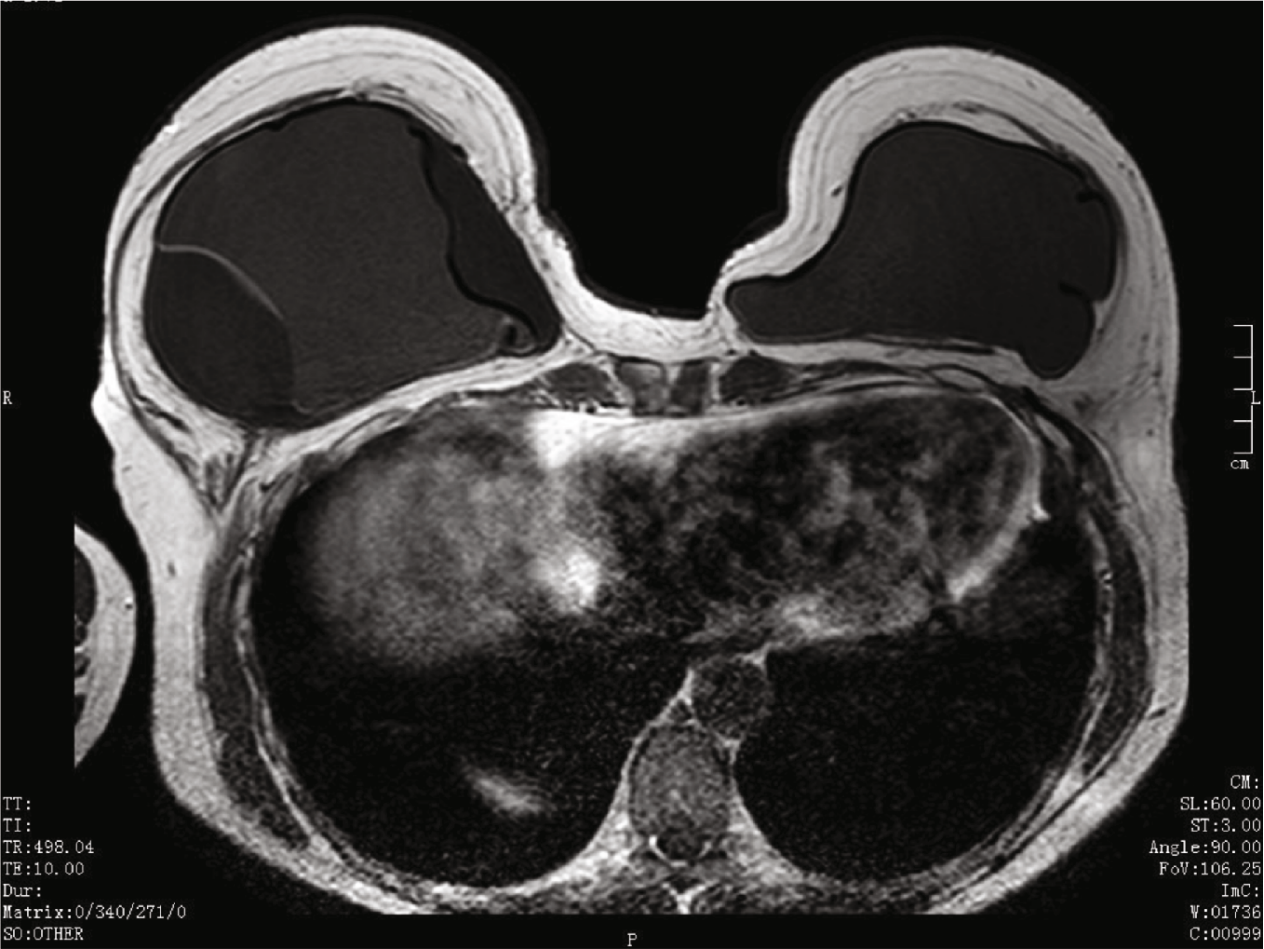
Breast MRI. Smooth circumferential thickening of the right implant fibrous capsule noted with diffuse progressive enhancement.

In July 2018, she was reviewed again in breast clinic and clinical concern persisted regarding the cause of the large seroma. It was felt that BIA-ALCL had not been completely excluded. An MRI scan was performed along with further ultrasound-guided aspiration to dryness (250 ml). The MRI showed smooth circumferential thickening of the right implant fibrous capsule with diffuse progressive enhancement (Figure 1). A moderate volume fluid collection was seen between the capsule and the implant shell, which was folded secondary to the fluid build-up. No implant rupture was evident. On the left side, the fibrous capsule was of normal thickness, with no enhancement and no intracapsular fluid collection. In view of her symptoms and the risk of breast implant-associated lymphoma, the patient was listed for a right-sided total capsulectomy and renewal of the right implant in September 2018.

**Figure 2:**
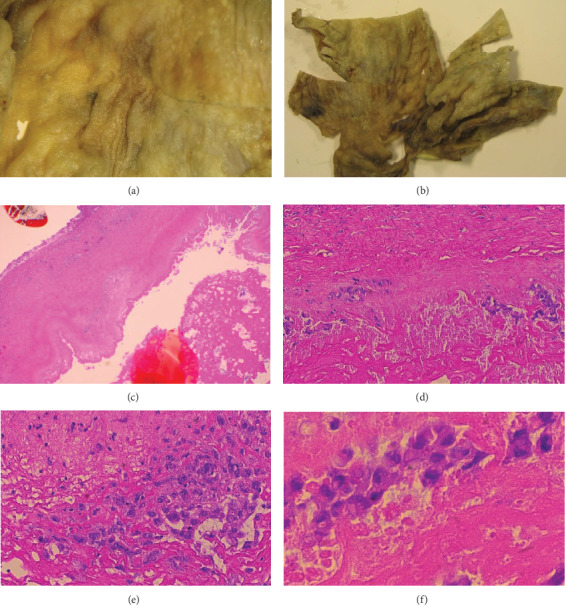
(a, b) Right breast capsule macroscopic appearance. Initial (malignant) capsulectomy specimen showing thickened, “velvety” appearance. (c–f) Right breast peri-implant capsule with exudate, microscopic appearance. Malignant lymphoid cells within the fibrinous exudate show large, irregular nuclei with prominent nucleoli.

**Figure 3:**
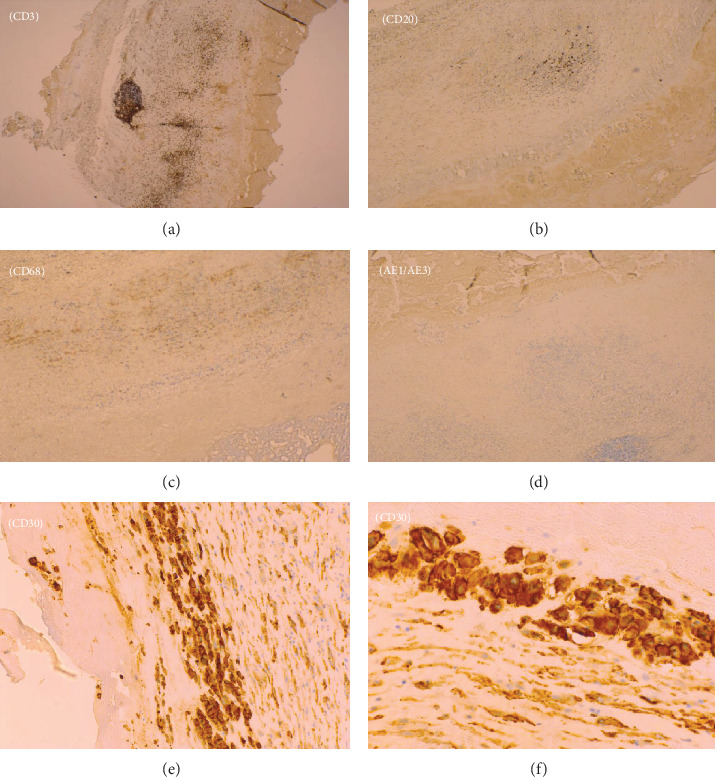
(a–f) Immunohistochemical staining of the right breast peri-implant capsule with exudate. The malignant lymphoid cells stain positively for CD30 and are negative for CD3, CD20, CD68, and AE1/3.

The right capsulectomy tissue was sent for pathological assessment. Marked thickening, fibrinous debris, and a “velvety” appearance of the capsular surface were noted (Figures 2(a) and 2(b)). Histological analysis showed that there were atypical lymphoid cells in the fibrinous exudate from around the implant (Figures 2(c)–2(f)). Tissue from the capsule was sent to HMDS for further analysis and second opinion. T-cell receptor (TCR) gamma multiplex polymerase chain reaction (PCR) showed an oligoclonal pattern of TCR rearrangement with multiple, reproducible, clonal populations. CD30 and IRF4 immunostains were positive. Alk, CD2, CD20, CD3, CD4, CD5, CD7, CD8, and Granzyme B were all negative (Figures 3(a)–3(f)). T-cell antigen expression was difficult to assess, but the features suggested a null phenotype. The overall pattern of morphology, immunohistochemistry, and molecular analysis was considered consistent with a diagnosis of breast implant-associated anaplastic large cell lymphoma (BIA-ALCL). At the time of diagnosis, the stage of the disease was stage 1B (T2N0M0) as per Clemens et al. [10].

In October 2018, she was seen again in breast clinic and further fluid accumulation was aspirated. A CT scan showed flattening of the new right breast prosthesis, with a surrounding effusion, abnormal soft tissue, and skin thickening, possibly representing postoperative changes. The patient was reviewed by the Manchester Lymphoma Group who advised urgent bilateral explant with capsulectomy or cavity shavings and salvage of any peri-implant seroma for full review with HMDS Leeds.

In November 2018, the patient was seen in breast clinic and expressed her preference for removal of the reconstructions, including implants and capsules, on both sides. Implant removal, total capsulectomy, and removal of an enlarged right axillary lymph node were performed. The tissue and fluid were sent for analysis.

No evidence of residual malignancy was seen in either the right or left breast capsulectomy/reconstruction tissue, peri-implant fluid, or the enlarged lymph node. Sections from the left breast tissue showed a mature peri-implant capsule with only focal sparse chronic inflammation, consistent with the number of years since reconstructive surgery on that side.

**Figure 4:**
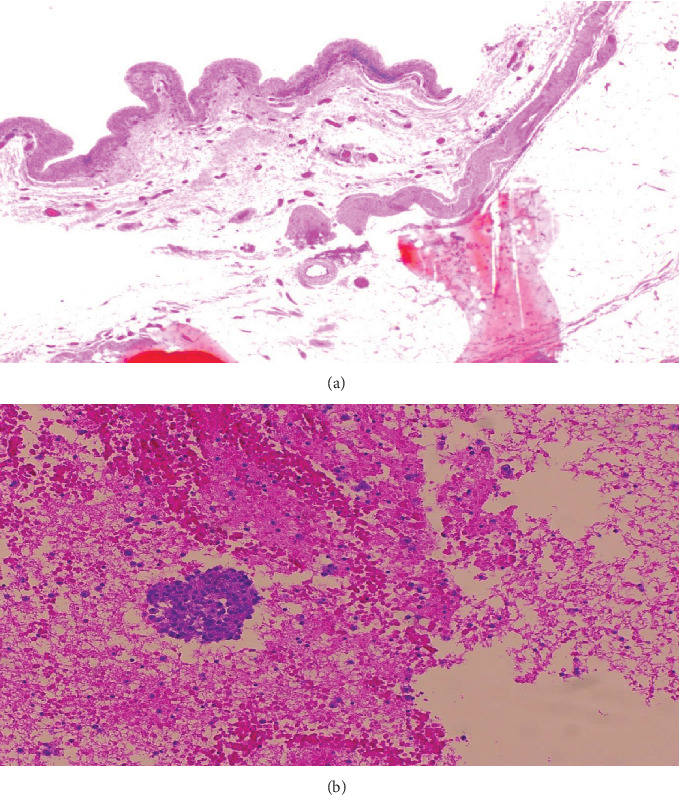
(a, b) Reresection of the right breast peri-implant capsule with exudate, microscopic appearance. Marked synovial metaplasia of the capsule and focal infiltration by small lymphocytes. No evidence of residual malignancy.

The right breast tissue included a newly formed capsule following the recent capsulectomy and implant renewal on that side. No nodules or masses were noted. Histological sections showed marked synovial metaplasia of the capsule and focal infiltration by small lymphocytes (Figures 4(a) and 4(b)). Aggregates of foamy histiocytes and foreign body giant cells were seen surrounding silicone droplets in the nearby adipose tissue, indicating mild silicone leakage. CD30 staining was negative.

## 3. Discussion

The World Health Organization provisionally classified BIA-ALCL in 2016. In the same year, a management algorithm was developed, in the United States, for the investigation and management of BIA-ALCL by the National Comprehensive Cancer Network [7, 10]. No official guidelines have been published within the United Kingdom.

With increasing recognition of BIA-ALCL, the recently launched Breast and Cosmetic Implant Registry (BCIR) will be ever more important, to allow accurate figures on implant use and the consequent follow-up of patient outcomes. Awareness and appreciation of this disease entity among clinicians are vital to ensure patients undergoing implant surgery are fully informed about BIA-ALCL [11] and consented to be included in the BCIR.

Recent theory has linked hyperplasia of T-cells to bacterial biofilms, as a possible cause of BIA-ALCL. Therefore, it is imperative to reduce infection risk, reducing chances of capsular contraction and subsequently reducing the activation of lymphocytes and possible BIA-ALCL conversion [12].

BIA-ALCL is not a disease of the breast parenchyma but is limited to the fibrous capsule that surrounds the implant. Clinical presentation is varied with the development of seroma and effusion being common, typically occurring between 1 and 7 years post reconstruction, as demonstrated in the above case. Nonspecific symptoms include redness, tenderness, and breast enlargement, but occasional patients present with a firm mass. As in our case, BIA-ALCL classically has overexpression of CD30 and is negative for Anaplastic Lymphoma Kinase (ALK) [2].

Non-BIA-associated ALCL is a very rare, aggressive form of lymphoma with poor prognosis. Traditionally, ALCL is staged using the Ann Arbor grading system. Clemens et al. have proposed a BIA-ALCL staging system, which identifies low-risk individuals with localised intraluminal disease and higher risk individuals with extraluminal disease, requiring more aggressive systemic therapy [9]. The case we describe appears to be within the low-risk cohort, with a good prognosis following surgical management. For patients diagnosed with BIA-ALCL, the overall survival rate is 89% at five years.

Our case demonstrated BIA-ALCL in initially negative cytology. If seroma is present, pathologists should be supplied with as much fluid as possible (minimum 20–50 ml; ideally >100 ml) in order to thoroughly evaluate and perform further tests such as flow cytometry and molecular studies, which may be necessary for diagnosis [7, 10]. Cytology specimens do not require any specialist media and should be transported within 48 hours. In our case, the fluid sample for cytology appears to have been only 10 ml in volume (with 30 ml of fixative), suggesting that undersampling may have been the reason for negativity. Certainly, diagnostic error seems very unlikely given that the specimen was reviewed by multiple pathologists, including at a specialist centre, and also underwent immunohistochemical testing.

Our case highlights that cytology sample volume is important in the investigation of patients with suspected BIA-ALCL. The case also emphasises that if diagnosis is indeterminate following initial analysis, then a secondary evaluation, potentially at a tertiary centre, may be necessary to come to a final diagnosis of, or confidently exclude, BIA-ALCL [9].

## 4. Conclusion

The case we describe highlights the importance of a high clinical index of suspicion for BIA-ALCL, even in cases where initial peri-implant fluid testing by cytology is negative. Peri-implant aspirations for cytology should comprise as large volume as possible, in order to maximise the likelihood of achieving a diagnosis and reduce false-negative results.

In our case, the initial surgical resection specimen provided both the diagnosis and the primary treatment for this unusual type of lymphoma. Following initial resection of the affected capsule, our patient opted for bilateral capsulectomy and removal of her breast reconstructions. Further research is needed to improve the evidence base in this area and refine surgical treatment guidelines.
